# An Account of the Good Effects of a Solution of Sal Ammoniac, in Vinegar, Employed, as a Topical Application, in Cases of Lacerated Wounds

**Published:** 1795

**Authors:** Henry Yates Carter

**Affiliations:** Surgeon at Kettley, Near Wellington, in Shropshire.


II. *An Account of the good Effects of a Solution of Sal Ammoniac, in Vinegar, employed, as a topical Application, in Caſes of lacerated Wounds. By Mr.* Henry Yates Carter, *Surgeon at Kettley, near Wellington, in Shropſhire.*

IN the ſecond volume of Medical Facts and Obſervations*, I took occaſion to mention, in a curſory manner, the good effects I had experienced, in lacerated wounds, from a ſolution of ſal ammoniac in vegetable acid, employed ployed as a topical application; and which, in fuch cafes, I obferved, had feemed to promote the union of the parts and to moderate the dif-charge. As this mode of treatment is very different from that commonly in ufe, and I have had occafion to try it in many cafes of bad compound fracture, and other lacerated wounds, in which there has been a tendency to fphace-lus, I have been induced to make it the fubject of a diftinct paper, and for this purpofe have felected the following cafes, from a greater number, in which I have ufed it; and thefe, I hope, may be deemed fufficiently interefting to procure their infertion in a future volume of the valuable collection above referred to.

### CASE I.

A poor man, named Ingram, aged upwards of eighty years, received an injury on his right foot, from a carriage paffing over and lacera-ting it from the inftep to the toes. The wound had been neglected for fome days, when I was requefted by a benevolent gentleman in the neighbourhood to vifit him, and found the foot

 fphace-

ſphacelated as high as the ancle, and the inflammation apparently extending ſtill farther.

I began with ſcarifying different parts of the foot, by which means I gave vent to a conſiderable quantity of acrid ichor. The whole foot was then well covered with lint, continued to ſome diſtance above the diſeaſe, and directed to be kept conſtantly wet with a mixture compoſed of half an ounce of crude ſal ammoniac diſſolved in a pint of vinegar. Internally he took the bark in ſubſtance, liberally, with opium, as he had a diſpoſition to diarrhœa.

On the ſecond day after this mode of treatment had been adopted, I had the ſatisfaction to find that the inflammation had not ſenſibly increaſed, and that the patient felt at intervals a throbbing, but which, he ſaid, was not painful, about the ancles. His pulſe, which had been much quicker, was now at 100.

On the ſixth day, a viſible ſeparation of the morbid parts was diſcoverable, and matter was perceptible on the verge of the ſeparating parts; a fluctuation was felt in ſeveral parts of the foot, particularly beneath thoſe places that had been ſcarified; and upon making deeper inciſions here, we diſcovered a collection of good pus and granulations of new fleſh. In the courſe courfe of a fortnight, the floughs, having previoufly become loofe, were gradually taken away, and the parts expofed one clear uniform wound. After this the bark was adminiftered lefs frequently, but the ufe of the lotion was continued till the wound was nearly healed, which happened in about two months.

### CASE II.

A girl, aged nineteen years, was attacked by a maftiff, and had the mufcles of the thigh and leg, particularly the vaftus externus and gaftrocnemius fo violently lacerated, that the worft confequences were to be expected from the circulation being cut off in the large veffels from the extremity, notwithftanding which fhe loft little or no blood; a circumftance, by the bye, that frequently occurs in lacerated wounds. She fuffered but little pain, although the feparated mufcles of the upper part appeared to be much irritated. The large portions of mufcle yet adhering were cautioufly replaced as near their original fituation as the nature of the cafe would admit; and after the parts had been well bathed with warm vinegar, and due proportions of lint applied round the limb, the whole was encompaſſed with a broad roller, applied merely tight enough to retain the dreſſings; the limb was then laid in an horizontal poſition, and the preſſure taken from the affected part by means of a pillow placed under the lower part of the leg, conſiderably below the injury. The whole was then wet with a lotion compoſed of half an ounce of crude ſal ammoniac diſſolved in a pint of vinegar, and ordered to be kept ſo conſtantly.

The firſt day ſhe was but little ſenſible of the application. At night a draught, containing twenty drops of laudanum, was given, and ſhe reſted well.

On the ſecond day I found her pulſe but little quickened, and her thirſt moderate; ſhe had perfect feeling in every part of the limb, and complained of an acute ſmarting in the wound upon every renewal of the lotion, which continued for a few minutes, and then ſhe became eaſy. An opening draught was given this morning, and ſhe repeated the opiate at night.

On the third day matter ſeemed to be forming, ing, but there was no appearance of inflammation or ſwelling of the limb.

On the fifth day from the receipt of the injury, the bandage was carefully removed, and I had the ſatisfaction to find that the muſcles had united, and that the parts of the bone that had been laid bare were covered with new fleſh. The diſcharge was kindly, and in moderate quantity, and the limb was free from pain. The ſame mode of dreſſing and the ſame applications were continued without alteration during three weeks, at the end of which time the cure was complete.

### CASE III.

A young man, aged nineteen years, by a fall of coal in the pit while he was ſtooping, was preſſed to the ground, and had his thigh broke about four fingers breadth above the patella. The upper part of the bone was forced through the muſcles and into the ground, ſo that the hollow of the bone was filled with dirt, and ſtripped bare nearly four inches, and the muſcles much lacerated. In this ſituation he was brought home, (about a mile) and I then faw him; the wound bled but little.

In this cafe I determined to try the effect of keeping the limb gently extended, nearly at its original length, after taking off fo much of the bone as I fhould find requifite to a complete and exact reduction and to get above the coal flack which had been introduced.

As the bone was fhivered longitudinally, I found it neceffary to take off about three inches of it. This being done, and the wound well cleanfed with warm vinegar and a fmall proportion of fpirit of wine, I placed the lower part of the limb as exactly parallel to the other as poffible, and retained it in that pofition by means of proper bolfters on each fide of the limb. An eighteen-tailed bandage having been previoufly laid under the part, the dreffing was made by gently filling the vacancy (the whole fide of the leg *externally* being open) with foft pledgets of lint dipped in the fame folution as that ufed in the preceding cafe, and the bandage was then applied as gently as poffible, in order to prevent the flefh from being preffed into the part that the bone ought to have occupied; and a fplint applied externally on each fide, merely to give more fteadinefs to the limb, but without occasioning much pressure. I think it right to mention also that the middle tails of the bandage were cut smaller than the others, and applied in such a manner that the wound might be uncovered, in order that the lotion might be applied immediately to the wound, without disturbing any other part.

He was let blood, and twenty-five drops of tincture of opium were given at night, and the attendant was strictly enjoined to keep the part constantly wet with the solution, except only during the intervals of sleep.

Upon visiting him the morning after the accident, I found he had had but little sleep, though his limb had given him but little pain, except for about a quarter of an hour after the application of the lotion, after which he said he had felt the whole leg and foot become sensibly warmer. The lower part of the limb lay very steady, exactly in the situation in which it had been placed; he took this morning three grains of calomel, which procured one stool.

On the fifth day, including the day of the receipt of the injury, (there having been some appearance of matter between the folds of the bandage) the dressings were wholly removed, and the wound was found covered with a wellconcocted concocted pus in moderate quantity, and with new granulations. The dreffings were continued in the fame manner as before, the whole vacancy being carefully filled with doffils of lint, made as foft as poffible, till the whole was level with the fkin; and over thefe the bandage was applied as before. He continued to repeat the opiate every night, and the calomel occafionally; his appetite was tolerably good, he ufed nearly the fame diet as when in health, and was permitted to drink a fmall quantity of ale.

On the eighth day the dreffings were again removed, and the appearances continued to be favourable. From this time, the weather being warm, the wound was dreffed every day in the fame manner as at firft; and in about eight weeks the callus was completely formed, and had filled up the void fpace, and the wound was reduced to about a quarter of an inch in diameter.

In ten weeks he came down ftairs, and went about on crutches; and in about fixteen weeks from the time he received the injury, he went with a ftick only, and was able to walk nearly two miles. The limb was not quite an inch fhorter than the other; the fmall ulcer continued nued to diſcharge, till a conſiderable exfoliation of bone, which gradually made its way outwards, was extracted, after which the wound ſoon healed.

### CASE IV.

A boy, aged about fifteen years, had the misfortune to ſlip his hand under the axletree of a water-wheel, which moves at about the diſtance of two inches and a half from a brick wall or buttreſs ſupporting another building; his arm was taken in to the elbow, and the machine performed ſeveral revolutions on the part before he could be extricated. The fleſh was ſtripped down on each ſide of the thick part of the arm, and the thumb was nearly ſeparated; but the fingers and hand had ſuffered but little, and there was no hæmorrhage. The thumb was not taken off, but carefully replaced, as well as the other muſcular parts that had been ſeparated; and to the whole wound a large quantity of lint was applied, wet with the ſolution of ſal ammoniac in vinegar. He took twenty drops of tincture of opium at night, but but he was very reſtleſs, and complained much of his arm.

Second day. The arm had bled in the night, and the dreſſings were become ſtiff and hard, which rendered it neceſſary to remove them. The diſturbance this occaſioned produced a degree of inflammation which, I believe, might otherwiſe have been prevented, and which proved the ſource of misfortune. The parts from this time became exceſſively painful, and the inflammation extended to the upper part of the arm, and to the ſhoulder and ſide, as far down as the pectoral muſcle. He was coſtive and feveriſh, and complained much of thirſt. The whole arm was wrapped in a cataplaſm made of oatmeal, with equal parts of vinegar and water; and three grains of calomel were immediately given. Two ſtools were procured by this medicine; but the pains ſtill continued to be very diſtreſſing to him. His pulſe was at 100.

Third day. The above ſymptoms continued; the pulſe was increaſed to 110; and he was at times delirious; the upper parts of the arm, ſhoulder, and ſide, were become of a dark red colour, and were exceedingly tenſe. He had ſeveral looſe ſtools; the arms and ſide were dreſſed dreſſed as before, with the addition, in the liquid of which the poultice was made, of half an ounce of crude ſal ammoniac, and an ounce of ſpirit of turpentine. He took half a drachm of Peruvian bark, with fifteen drops of tincture of opium, every third hour; and care was taken to diſtil ſome of the ſolution between the dreſſings, upon the ſhoulder, very often, in ſuch a manner that it might make its way to the affected parts.

Fourth day. I found the whole fore arm, from the elbow, completely ſphacelated and dry; but the ſhoulder and ſide were nearly in the ſame ſtate as yeſterday, the inflammation not having increaſed; his purging had ceaſed; he was not ſo thirſty, and his pulſe was at 100; but he complained much of head-ach and wearineſs. Notwithſtanding there appeared ſome reaſon to conclude that his head-ach might, in ſome meaſure, be occaſioned by the quantity of opium he had taken, I continued the uſe of it in the ſame doſes; a ſtool was procured by means of a clyſter. The uſe of the lotion was continued.

Fifth day. The ſymptoms were nearly the ſame as yeſterday. The ſame dreſſings and medicines were continued.

Sixth

Sixth day. The pain and tenſion were much leſſened, he had reſted tolerably well, and was free from thirſt; the ſhoulder and ſide, with a conſiderable part of the upper arm, ſeemed approaching to their natural colour, and the extent of inflammation was viſibly decreaſing. The bark was ſtill continued, but without the tincture of opium, inſtead of which he took two grains of purified opium at night.

The cataplaſm was continued as before for about a week, from this time, when the ſhoulder and ſide having recovered their original tone, it was changed for one compoſed of oatmeal and the ſolution alone. In a few days matter formed plentifully round the bone in thoſe parts where the lacerations had been deep, and large portions of the muſcles were cautiouſly removed. The matter formed was of a good conſiſtence, and moderate in quantity; and the wound was perfectly eaſy, excepting only upon the application of the lotion, and for ſome ſhort time after. The whole hand dropped off at the wriſt; the other parts gradually filled up with good fleſh, and are now completely healed.

### CASE V.

A man, aged thirty-ſix years, by the fall of a very heavy iron rod perpendicularly upon his foot, upon that part where the ſhoe is generally buckled, received a conſiderable lacerated wound, by which the tendons were much injured, and the integuments and muſcular fleſh were ſtripped off from the upper part of the tarſus, and hung in a large looſe flap down the ſide of the foot. The wound bled conſiderably, and the whole foot, from the violence of the blow, was inſenſible. The parts were well cleanſed from the grumous blood with vinegar and water, with a ſmall quantity of ſpirit of wine, and the looſe flap replaced in the ſituation from which it had been torn, and dreſſed with pledgets of lint dipped in the ſolution; and a cataplaſm applied of oatmeal and vinegar.

The morning after the injury, upon removing the dreſſings, the wound and whole foot were found to have a favourable appearance; but at night he began to complain of a great degree of heat, throbbing, and ſenſe of tenſion.

On

On the third day, on removing the dreſſings, the whole upper part of the foot appeared to be haſtily approaching to a ſphacelated ſtate. It had loſt all ſenſibility to the touch, and the inflammation had increaſed, though in ſo ſhort a time, conſiderably above the ancle, and to the extremity of the toes. A ſenſation of burning heat in the whole foot and leg ſtill continued. The parts that were looſe were now removed, and the wound, after having been bathed a conſiderable time with a mixture of warm vinegar and water, with a ſmall quantity of ſal ammoniac previouſly diſſolved in it, was dreſſed as uſual, the lint being firſt well ſaturated with the lotion; and over the whole a cataplaſm was applied as before. A purgative medicine, compoſed of four grains of calomel, and five grains of aloes, was given, which operated well. He paſſed this day with ſomewhat more eaſe, and at night took thirty drops of tincture of opium.

Fourth day. He complained of having paſſed a very reſtleſs night, and that the painful ſenſation of burning heat ſtill continued; the inflammation went on increaſing; his pulſe was at 97, and he had much thirſt and fluſhing heat. Bark, in the quantity of half a drachm, was was given every third hour, and twenty drops of tincture of opium every ſixth hour. The ſame dreſſings were continued, with the poultice; but at night the poultice was omitted, and the dreſſings kept wet with the ſolution alone.

Fifth day. He had reſted much better; his thirſt was more tolerable, and the heat and other ſymptoms were much more moderate; his pulſe was at 90; the inflammation had not increaſed; and the tenſion about the ancle was leſſened. The ſame medicines and local applications were continued as laſt night. On renewing the dreſſings in the evening, he complained of having paſſed a very painful afternoon, and that the ſenſe of heat had been greater. He attributed all this to the omiſſion of the poultice, which was now, at his earneſt requeſt, renewed.

Sixth day. In the morning the ſymptoms were much increaſed, and the inflammation was ſpreading, with a violent degree of pain and tenſion, the whole upper part of the foot being in a ſphacelated ſtate; and the patient complained of exceſſive pain. The ſame dreſſings as before were applied, but without the poultice, after bathing the parts with warm vinegar; a broad roller, for the convenience of keeping the parts wet, was gently applied over all the inflamed parts; and as I had a ſuſpicion that the increaſe of his pain, &c. yeſterday, if not wholly, was, in a great meaſure, owing rather to a want of due care in keeping the parts conſtantly moiſt, and thus ſuffering them to get dry and hard, than to any effect the application could have in producing thoſe ſymptoms, I paid this day a particular attention to this circumſtance, by viſiting him ſeveral times, to ſee that the ſolution was duly applied; and in a few hours the ſymptoms of pain and heat in the whole limb were greatly diminiſhed, and continued gradually to abate the whole day. His pulſe at night was at 93.

Seventh day. The ſymptoms were nearly the ſame as yeſterday; the inflammation, upon the whole, was rather leſs, but there was no appearance of matter. He had paſſed a tolerable night; but his pulſe was ſtill at 93. As he was coſtive, the purgative medicine was repeated.

Eighth day. He had paſt a good night, comparatively ſpeaking; the pain in the upper part of the limb (or above the diſeaſe) was conſiderably leſſened, and the inflammation was much leſs; a ſmall quantity of matter appeared upon the edges of the lacerated parts; his pulfe was at 90. He began to complain of fevere fmarting upon the renewal of the lotion, and at times infifted on its application being deferred to longer intervals, though when the parts began to grow dry, the heat and fenfe of ftricture were conftantly renewed.

Ninth day. He had paffed a reftlefs and painful night; his foot and leg were in much pain at intervals, but (exclufive of the fmarting pain for a quarter of an hour upon the lotion being applied) he always became much eafier after the wetting of the parts, which took place once in about two hours, unlefs fleep intervened.

From this time the ufe of the lotion was continued in the fame manner as before, and he continued alfo to perfevere in the ufe of the bark and opium; the floughs feparated kindly; the inflammation went off from the leg and toes, and a feparation of the difeafed parts took place at a very little diftance from the edges of the original injury. The wound difcharged a well-formed matter, and as the parts beneath fome of the thickeft floughs granulated, the latter gradually came away without much pain, and the whole was healed in ten weeks, except a very fmall ulcer upon the lower part of the Tarfus, through which a fmall exfoliation made its way.

As in the preceding cafes I was careful to obviate the effects of irritation, by keeping the bowels moderately open, giving occafionally, and fometimes liberally, of opium; and invigorating the fyftem by means of wine and the Peruvian bark; it may perhaps be fuggefted, by fome readers, that the favourable termination of the cafes I have been relating was due rather to the internal than external remedies employed; and that to fubject to a fair and decifive trial this or any other remedy, no other fhould be employed at the fame time. This is indeed what I have done in flighter cafes of laceration, where local applications only were requifite; and in all fuch cafes the union of the parts has appeared to me to be much more fpeedily effected by means of the lotion, than it is by the ordinary mode of treatment. And I am able to recollect no inftance of bad compound fracture, or of lacerated wounds, attended with or threatening fphacelus, where the warm fomentations and cataplafms commonly employed in fuch cafes were were made ufe of, in which there was any fuch obvioufly good effect from the local treatment, as in the cafes I have been defcribing; notwithftanding there was the fame liberal ufe of opium and Peruvian bark, &c. internally. On the contrary, I have but too often feen the worft effects from fuch cataplafms, &c.; and in one of the above cafes, (Cafe V.), the bad effects of a poultice, applied at the earneft requeft of the patient, were very ftriking, when contrafted with the relief he afterwards experienced from the ufe of the lotion.

